# Isolated Bilateral Abducens Palsy as a Presenting Feature of Multi-Infarct State: A Case Report

**DOI:** 10.7759/cureus.21286

**Published:** 2022-01-16

**Authors:** Saurabh Kumar, Bharat Seju, Durga Shankar Meena, Arjun Kachawaha, Maya Gopalakrishanan

**Affiliations:** 1 Internal Medicine, All India Institute of Medical Sciences, Jodhpur, IND

**Keywords:** acute infarct, stroke, abducens nerve, isolated, cranial nerve

## Abstract

Isolated abducens palsy is a rare clinical entity. The usual causes of bilateral sixth nerve palsy are head trauma, tumor (skull base), aneurysm, and ischemic stroke. Bilateral abducens palsy without any other neurological deficit secondary to ischemic stroke is a rare clinical presentation. We present a case of a 78-year-old male without any comorbidities with a history of diplopia for the last two months. Physical examination was unremarkable except for bilateral sixth nerve palsy. MRI brain showed the chronic ischemic area in the pons, bilateral basal ganglia, deep white matter, and periventricular region of bilateral frontal, temporal, parietal, and occipital lobe. This report highlights an unusual presentation of ischemic stroke as isolated bilateral abducens palsy without any other focal neurological deficit.

## Introduction

Isolated bilateral sixth nerve palsy is an uncommon presentation in the emergency department. The common causes are acquired, located along its course (between its nuclei in pons to lateral rectus muscle in orbit). Due to its long extracerebral intracranial course, the abducens nerve is vulnerable to injury in various disorders. Bilateral sixth nerve palsy is found in only 10% of cases in the literature [1]. Patients presenting with bilateral sixth nerve palsy need evaluation for raised intracranial tension, mass lesions, trauma, demyelinating diseases, and underlying vasculitis. There are reports describing isolated bilateral sixth nerve palsy in cases of trauma, aneurysmal rupture, and temporal arteritis [2-4]. However, a recurrent stroke with bilateral pontine infarct presenting as isolated bilateral sixth nerve palsy is rare. To the best of our knowledge, there is no reported case so far where isolated bilateral sixth nerve palsy is the presenting feature of recurrent stroke.

## Case presentation

**Figure 1:**
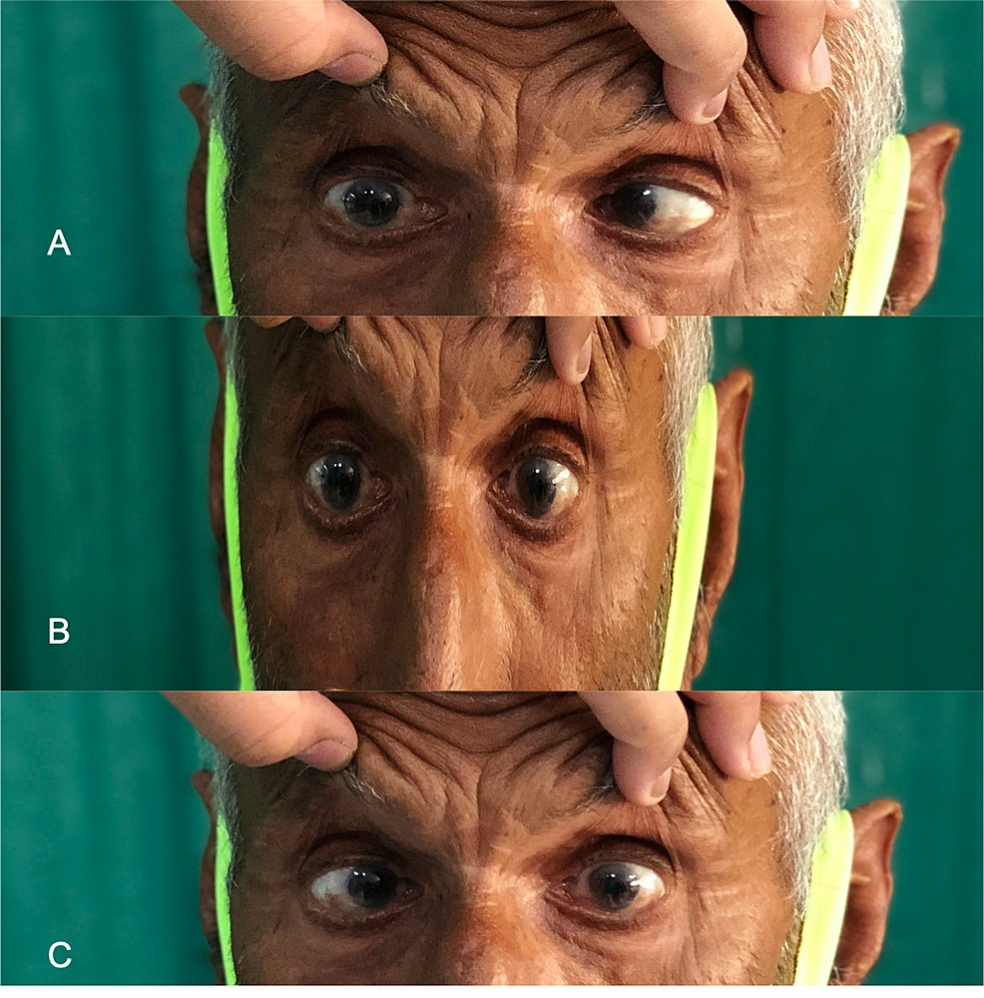
Image showing limitation in the abduction of bilateral eyes ({A} right eye in lateral gaze, {B} primary gaze, and {C} left eye in lateral gaze) suggestive of bilateral abducens palsy.

A 78-year-old male farmer from western Rajasthan, without any comorbidities or addiction, presented with a complaint of neck pain for the last six months. The neck pain was radiating to the bilateral upper arm and head. For the previous two months, he has been complaining of double vision from the left eye, which was sudden in onset, and for the last one month, he has had double vision from both eyes. There was no history of weight loss, anorexia, eye pain, altered sensorium, fever, vomiting, dizziness, difficulty in walking or handling things, or slurring of speech. On examination, pulse rate was 84/min regular, and blood pressure was 124/68 mmHg in the right brachial artery. Bilateral lateral rectus muscle palsy was present (Figure 1).

The rest cranial nerves were normal in the examination. No other focal neurological deficit was found. Bilateral plantar were flexor. No carotid bruit was appreciated. The temporal artery was non-palpable; in addition, erythrocyte sedimentation rate (ESR) was 43 mm in the first hour. There was no evidence of papilledema on fundoscopy. Lumbar puncture was done, cerebrospinal fluid (CSF) pressure was 8 mmHg. CSF glucose was 68 mg/dL (plasma glucose level was 98 mg/dL), the protein was 44 mg/dL, and adenosine deaminase (ADA) was 4 U/L. CSF cytology showed five white blood cells (lymphomononuclear) and two red blood cells (RBC) without any atypical cells. CSF Gram stain did not reveal any microorganisms. No acid-fast bacilli were seen on Ziehl-Neelsen stain; no capsulated organism was appreciated on India ink. CSF aerobic culture and sensitivity were negative for the growth of any organism. CSF for mycobacterial culture also came negative. Magnetic resonance imaging of the brain with the spine was done, which was suggestive of the chronic ischemic area in the pons, bilateral basal ganglia, deep white matter, and periventricular region of bilateral frontal, temporal, parietal, and occipital lobe. The MRI spine was suggestive of degenerative changes in the cervical spine. Two-dimensional echocardiography (2D echo) was done to rule out cardioembolic stroke, which showed normal chambers size with 60% ejection fraction; no clots or vegetations were appreciated. CT angiography of neck and brain was done, which showed hypoplastic right vertebral artery with normal left vertebral artery. Based on these findings and after ruling out all causes of increased intracranial pressure (ICP) and bilateral cranial nerve palsy, the patient was diagnosed with ischemic stroke and was started on antiplatelets, statins for secondary prevention, and is doing so well on follow-up with ocular rehabilitation and physiotherapy.

## Discussion

The abducens nerve nucleus is present in the dorsal pons and responsible for horizontal gaze. It has the longest intradural course. Hence it is the most common nerve involved in increased intracranial pressure (ICP) [5]. Abducens palsy is classified as isolated (no associated neurological deficit present) or non-isolated (associated with other neurological deficits, including other cranial nerve palsies). According to a recent study, the incidence of sixth nerve palsy is 4.66 per 100,000 people per year, with an increase in overall incidence in the elderly population (peak incidence at 65-69 years of age in males and 70-74 years of age in females) [6]. Isolated abducens palsy may be congenital, traumatic, post-viral, microvascular, or idiopathic [4,7-9]. Bilateral abducens palsy has diverse etiologies like increased ICP, Wernicke-Korsakoff syndrome, Guillain-Barre syndrome, giant cell arteritis, and post-operative or iatrogenic [2,10]. There are reports of multiple sclerosis presenting as isolated abducens nerve palsy [11,12]. Numerous studies have explored the etiology of abducens palsy with common causes remaining neoplasm (13-39%) followed by trauma (12-32%). In most studies, the proportion of cases with undetermined causes was relatively high (6-29%). Ischemia as a cause of abducent palsy was found in 8-36% of the patients [6]. Brain stem infarcts are more likely to cause unilateral abducens palsy (31%) than bilateral abducens palsy (6%). Few reports described unilateral abducens palsy secondary to pontomedullary infarction [13,14].

Horizontal diplopia is a common symptom in patients with isolated sixth nerve palsy. Every patient with bilateral abducens palsy should undergo a fundoscopy followed by CSF pressure measurement to rule out increased intracranial pressure. We did fundoscopy followed by a lumbar puncture which was unremarkable. Since the patient had a headache, we ruled out temporal arteritis as no temporal artery was palpable and ESR was in the normal range. Infective etiologies were ruled out by CSF examination, which showed normal biochemical, cytology, and microbiological parameters. It is imperative to rule out medullary infarct in such patients; there are reports describing sudden death in these patients without any specific neurological signs [15,16].

## Conclusions

Isolated bilateral abducens palsy is one of the rare presentations of ischemic stroke which can be misdiagnosed in emergency settings. Ischemic causes must be considered and managed in these settings after ruling out raised common causes like raised intracranial tension and trauma.
